# Childhood cancer incidence by ethnic group in England, 2001–2007: a descriptive epidemiological study

**DOI:** 10.1186/s12885-017-3551-7

**Published:** 2017-08-25

**Authors:** Shameq Sayeed, Isobel Barnes, Raghib Ali

**Affiliations:** 1 Cancer Epidemiology Unit, University of Oxford, Richard Doll Building, Oxford, OX3 7LF UK; 2 Public Health Research Center, New York University Abu Dhabi , Abu Dhabi, United Arab Emirates

**Keywords:** Childhood cancer incidence, England, Ethnic minorities

## Abstract

**Background:**

After the first year of life, cancers are the commonest cause of death in children. Incidence rates vary between ethnic groups, and recent advances in data linkage allow for a more accurate estimation of these variations. Identifying such differences may help identify potential risk or protective factors for certain childhood cancers. This study thus aims to ascertain whether such differences do indeed exist using nationwide data across seven years, as have previously been described in adult cancers.

**Methods:**

We obtained data for all cancer registrations for children (aged 0–14) in England from January 2001 to December 2007. Ethnicity (self-assigned) was established through record linkage to the Hospital Episodes Statistics database or cancer registry data. Cancers were classified morphologically according to the International Classification of Childhood Cancer into four groups – leukaemias; lymphomas; central nervous system; and other solid tumours. Age standardised incidence rates were estimated for each ethnic group, as well as incidence rate ratios comparing each individual ethnic group (Indian, Pakistani, Bangladeshi, Black African, Black Carribean, Chinese) to Whites, adjusting for sex, age and deprivation.

**Results:**

The majority of children in the study are UK born. Black children (RR = 1.18, 99% CI: 1.01–1.39), and amongst South Asians, Pakistani children (RR = 1.19, 99% CI: 1.02–1.39) appear to have an increased risk of all cancers. There is an increased risk of leukaemia in South Asians (RR = 1.31, 99% CI: 1.08–1.58), and of lymphoma in Black (RR = 1.72, 99% CI: 1.13–2.63) and South Asian children (RR = 1.51, 99% CI: 1.10–2.06). South Asians appear to have a decreased risk of CNS cancers (RR = 0.71, 99% CI: 0.54–0.95).

**Conclusions:**

In the tradition of past migrant studies, such descriptive studies within ethnic minority groups permit a better understanding of disease incidence within the population, but also allow for the generation of hypotheses to begin to understand why such differences might exist. Though a major cause of mortality in this age group, childhood cancer remains a relatively rare disease; however, the methods used here have permitted the first nationwide estimation of childhood cancer by individual ethnic group.

**Electronic supplementary material:**

The online version of this article (doi:10.1186/s12885-017-3551-7) contains supplementary material, which is available to authorized users.

## Background

In 2009, Cancer Research UK (CRUK) published Cancer Incidence and Survival By Major Ethnic Group for England 2002–2006 [1], linking incidence and mortality data from cancer registries with (self-assigned) ethnicity from the Hospital Episodes Statistics (HES) database. This methodology allowed a much more accurate estimation of outcomes by ethnic group and confirmed differences in incidence and survival in many of the different cancer types, with CRUK concluding that these differences needed ‘investigating further and the analyses extended’.

We have since published a series of papers to do that by looking more closely at individual ethnic groups and their differences in cancer incidence. South Asians and Blacks are not homogenous groups, with the subgroups within these broad categorisations having differing religious, social and cultural practices. We thus analysed cancer incidence in gastrointestinal [2], haematological [3], thyroid [4], breast and gynaecological [5], urological [6] and CNS [7] malignancies nationwide, looking individually at the difference between British Indians, Pakistanis, Bangladeshis (‘South Asians’), Black Africans, Black Carribeans (‘Blacks’) and Whites. These consistently show differences in incidence between the ethnic groups in many cancers; interestingly, they also suggest that these differences – between British Whites and ethnic minorities - can become less marked in some cancers over time [2], in keeping with previous studies in migrant populations [8] and suggesting possible environmental risk and protective factors where such patterns are observed over the space of a few generations.

Whilst some of these differences can be accounted for through known risk factors, there are many for which we do not currently have any good explanation. Thus, accurately confirming these ethnic differences (and related outcomes) through linked data, and using self-assigned ethnicity as the current most accurate measure of ethnicity [9, 10], allows not only for targeted public health spending and interventions, but is also a first step in attempting to identify potentially modifiable risk factors.

Beyond the first year of life, cancer is the commonest cause of death in childhood (ages 0–14) in England and Wales [11]. Whilst the CRUK report did not study this, here we consider for the first time the nationwide data for childhood cancer (2001–2007), using self-assigned ethnicity data and widening the analysis to include all of the above mentioned ethnic subgroups.

## Methods

The methods used in this study were broadly the same as those described in our previous studies [2–7, 12].

### Data collection

The National Cancer Intelligence Network (NCIN) provided data for all cancer registrations from January 2001 to December 2007 in residents of England aged 0 to 14 years old. For each registration, the following information was given: cancer site coded to the International Classification of Diseases, 10th Revision (ICD-10) [13]; morphology coded to the International Classification of Diseases of Oncology, 2nd and 3rd Revisions (ICD-O-2 and ICD-O-3) [14, 15]; deprivation assessed from the income domain of the Index of Multiple Deprivation 2007 (IMD 2007) [16]; age at diagnosis of cancer; sex and ethnicity. We used the mid-year population estimates produced by the Office of National Statistics (ONS) from 2001 to 2007, stratified by age, sex and ethnicity. Population data stratified by national quintiles of the income domain were provided by ONS based on the 2001 census and the same distributions applied to population data by age, sex and ethnicity for the 2001–2007 mid-year population estimates.

### Classification of ethnicity

NCIN obtained the self-assigned ethnicity for each cancer registration by record linkage to the HES database. If a cancer registration could not be linked or if ethnicity was missing on the HES database, then ethnicity was assigned using the cancer registry data. Prior to April 2001, ethnicity was classified by HES and the cancer registries according to the codes used in the 1991 census. After April 2001, the codes were amended to those used in the 2001 census, although 1991 ethnicity codes were accepted until 2003. For the analyses presented in this paper, ethnicity was classified as White (White from the 1991 Census and White British from the 2001 Census), Indian, Pakistani, Bangladeshi (with the three groups combined to form the category of ‘South Asian’), Black African, Black Caribbean (again both combined to form the category ‘Black’) and Chinese.

### Classification of cancers

We used morphology to classify cancers according to the International Classification of Childhood Cancer (ICCC-3) [17]. To do this we converted ICD-O codes from the second to third edition as necessary. As in previous studies [18], we classified cancers into four groups corresponding to the diagnostic groups I, II, III and IV-XII of the ICCC-3. These groups are respectively: leukaemias and myloproliferative and myelodysplastic diseases; lymphomas and reticuloendothelial neoplasms; central nervous system and intracranial and intraspinal neoplasms; and other solid tumours.

### Statistical analyses

We estimated age standardized rates (ASRs) of cancer per 100,000 person-years for all ethnic groups using direct standardization to the 1960 Segi world population [19], with age at diagnosis of cancer being classified into three categories: 0–4, 5–9, and 10–14 years. We used Poisson regression to estimate incidence rate ratios (IRRs) comparing each ethnic group (and the two combined groups, South Asians and Blacks) to Whites adjusting for sex, age and deprivation.

When comparing South Asians and Blacks to Whites, we present results as IRRs and 99% confidence intervals (CIs). When comparing the individual ethnic groups, results are presented as IRRs and 99% floating confidence intervals (FCIs). FCIs were calculated using the method of floating absolute risks [20, 21] and enable valid comparisons between any two ethnic groups, even if neither one is the baseline. We calculated 99% CIs because of multiple tests performed across ethnic groups. Tests of heterogeneity of IRRs between ethnicities, either overall or restricted to South Asians or Blacks, were performed using likelihood χ^2^ ratio tests.

We performed pre-specified subgroup analyses by sex. Tests of heterogeneity of IRRs between subgroups were performed for South Asians, Blacks and Chinese using a χ^2^ contrast test.

Because ethnicity information was not complete for all registered cancers, we performed a sensitivity analysis using multiple imputations of the missing ethnicity values based on age, sex, income and site of cancer.

We performed all analyses using Stata V.12 and R statistical software packages [22, 23].

### Graphical presentation of results

Where results are presented in the form of plots, we represent IRRs for each ethnic group by squares and their corresponding 99% FCIs by straight lines. For the combined South Asian and Black groups, we show IRRs as open diamonds, whose horizontal extent indicates the 99% CI. We placed dashed vertical lines at the value of the IRRs for South Asians and Blacks.

## Results

Demographic information for children in England from the 2001 Census is presented in Table 1. The total childhood population in England was 9,277,814 of which the majority (84.2%) were White.

**Table 1:** Comparison of demographics for children from major ethnic groups within the UK

Ethnic group	White		Indian		Pakistani		Bangladeshi		Black African		Black Caribbean		Chinese		Other Ethnicity	
	*N*	(%)	*N*	(%)	*N*	(%)	*N*	(%)	*N*	(%)	*N*	(%)	*N*	(%)	*N*	(%)
Census data for 2001																
Total population	7,812,159	(84.2)	218,508	(2.4)	232,507	(2.5)	99,713	(1.1)	136,170	(1.5)	106,616	(1.1)	36,523	(0.4)	635,618	(6.9)
Sex																
Male	4,005,190	(51.3)	111,778	(51.2)	118,661	(51.0)	50,691	(50.8)	68,602	(50.4)	53,423	(50.1)	18,507	(50.7)	324,055	(51.0)
Female	3,806,969	(48.7)	106,730	(48.8)	113,846	(49.0)	49,022	(49.2)	67,568	(49.6)	53,193	(49.9)	18,016	(49.3)	311,563	(49.0)
Age																
0–4	2,416,850	(30.9)	67,805	(31.0)	83,949	(36.1)	36,154	(36.3)	50,484	(37.1)	32,135	(30.1)	10,356	(28.4)	228,505	(36.0)
5–9	2,638,626	(33.8)	71,642	(32.8)	76,931	(33.1)	32,206	(32.3)	46,081	(33.8)	35,661	(33.4)	11,345	(31.1)	210,037	(33.0)
10–14	2,756,683	(35.3)	79,061	(36.2)	71,627	(30.8)	31,353	(31.4)	39,605	(29.1)	38,820	(36.4)	14,822	(40.6)	197,076	(31.0)
Deprivation																
Low	1,557,414	(19.9)	81,580	(37.3)	158,961	(68.4)	75,330	(75.5)	87,592	(64.3)	60,267	(56.5)	9023	(24.7)	221,578	(34.9)
Middle	4,622,489	(59.2)	113,946	(52.1)	66,152	(28.5)	22,574	(22.6)	44,348	(32.6)	43,320	(40.6)	20,051	(54.9)	314,577	(49.5)
High	1,632,256	(20.9)	22,982	(10.5)	7394	(3.2)	1809	(1.8)	4230	(3.1)	3029	(2.8)	7449	(20.4)	99,463	(15.6)
Country of birth: UK	*	*	202,371	(92.6)	211,770	(91.1)	88,068	(88.3)	92,266	(67.8)	99,095	(92.9)	28,963	(79.3)	.	.
Other	*	*	16,137	(7.4)	20,737	(8.9)	11,645	(11.7)	43,904	(32.2)	7521	(7.1)	7560	(20.7)	.	.

*Data unavailable

There is a greater proportion of older children amongst Whites, Indians, Black Carribeans and Chinese, with the reverse being seen in Pakistanis, Bangladeshis and Black Africans. Levels of deprivation also differed with the majority of Pakistanis, Bangladeshis, and Blacks having low incomes and the remaining ethnic groups being mostly middle or high income.

The majority of children were UK born, though the proportion varies between different ethnic groups from 68% in Black Africans to 93% in Black Carribeans and Indians.

### Comparing cancer incidence between ethnic groups

The total number of cancers in each ethic group is presented in Table 2, and analyses comparing the relative frequency (rates) of these cancers by ethnic group are presented graphically (see Figures).

**Table 2:** Number of cases and distribution of cancers across ethnic groups

	White	Indian	Pakistani	Bangladeshi	Black African	Black Caribbean	Chinese	All other ethnicities	No ethnicity recorded		Total
	*N*	*N*	*N*	*N*	*N*	*N*	*N*	*N*	*N*	(%)	*N*
Leukaemias	2329	72	115	34	35	28	10	376	224	(7.0)	3223
Lymphomas & reticuloendothelial neoplasms	761	34	33	13	37	4	2	138	137	(11.8)	1159
CNS & intracranial & intraspinal neoplasms	1694	46	38	7	36	30	3	255	234	(10.0)	2343
Other solid tumors	2739	49	91	26	84	37	25	425	459	(11.7)	3935
All cancers	7523	201	277	80	192	99	40	1194	1054	(9.9)	10,660

Leukaemias, then CNS cancers were the commonest in most ethnic groups except in Black Africans who had a similar absolute number of leukaemias, lymphomas and CNS cancers.

All analyses (Figures) are relative to Whites as the baseline group.

**Fig. 1:**
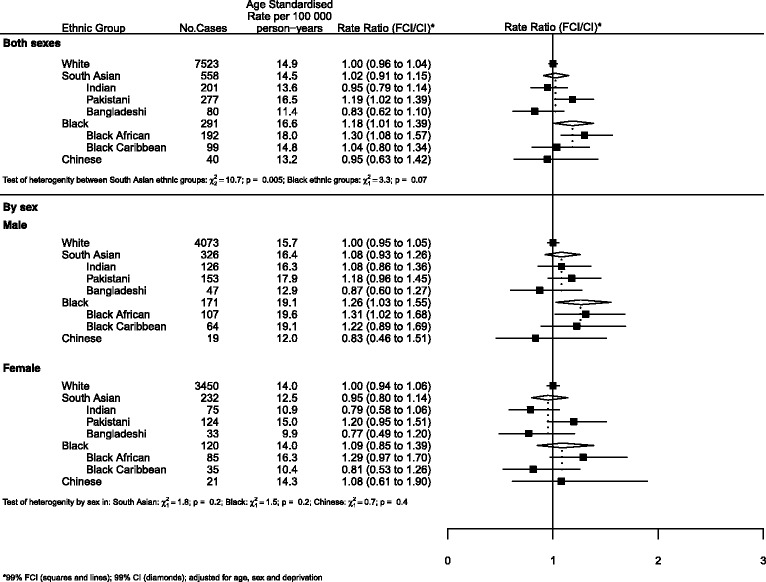
All cancers by ethnicity and sex

For all cancers (Fig. 1), there was little difference in risk between South Asians and Whites. However, there was strong evidence of heterogeneity within the group with Pakistanis at greater risk than Indians or Bangladeshis (RRs of 1.19, 0.95 and 0.83 respectively, *p* = 0.005). Risks among Blacks were higher than those of Whites, with no difference observed between Black Africans and Black Caribbeans.

**Fig. 2:**
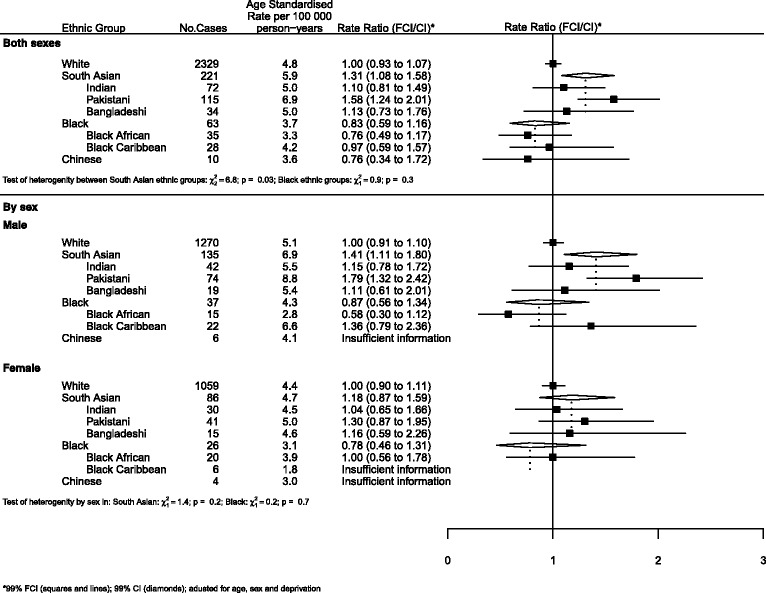
Leukaemias by ethnicity and sex

For leukaemias (Fig. 2), the risk among South Asians was approximately 30% higher than that of Whites. Again, there was evidence of heterogeneity within this group with Pakistanis at greater risk than Indians or Bangladeshis (RRs of 1.58, 1.20 and 1.13 respectively, *p* = 0.03).

**Fig. 3:**
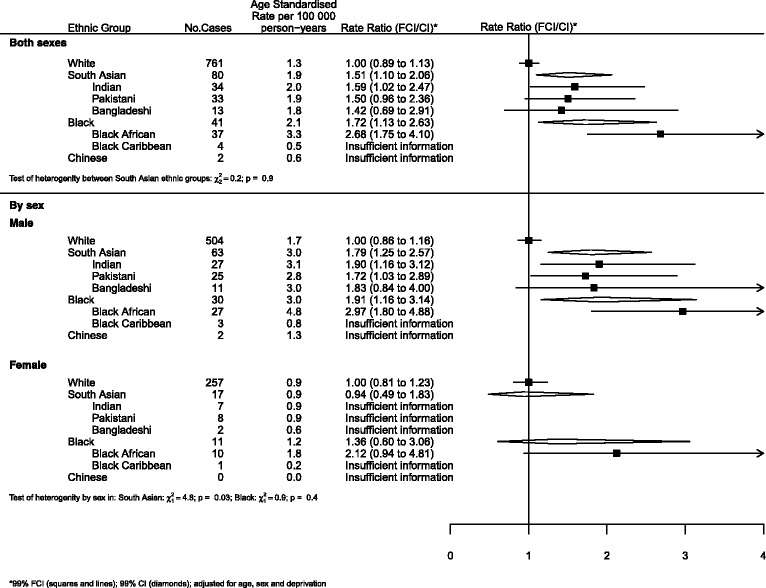
Lymphomas by ethnicity and sex

For lymphomas and reticulendothelial neoplasms (Fig. 3), both South Asians and Blacks were at increased risk. The risk for South Asians was approximately 50% higher than Whites and there was little evidence of heterogeneity within this group. The risk for Blacks was approximately 75% higher than Whites; there was insufficient information to examine heterogeneity within this group. Subgroup analysis revealed evidence of heterogeneity by sex in South Asians; the relative risk for males was higher than for females (RRs of 1.79 and 0.94 respectively, *p* = 0.03).

**Fig. 4:**
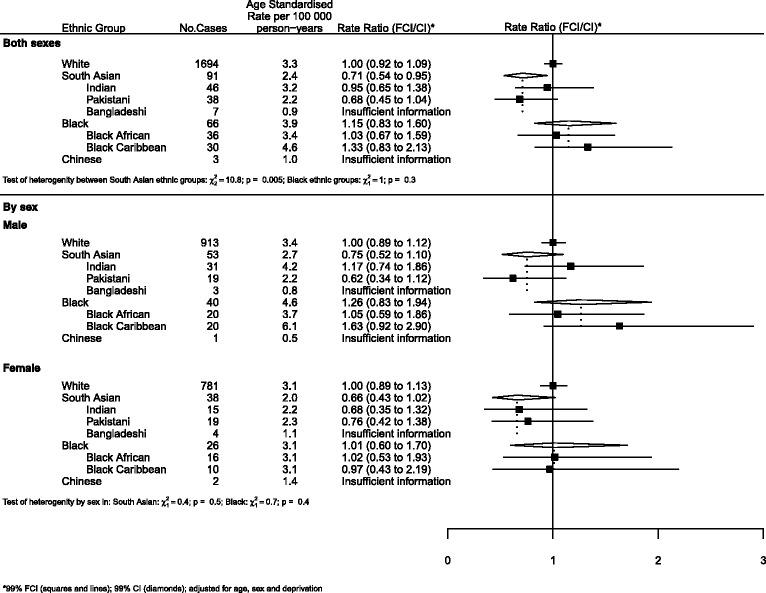
CNS cancers by ethnicity and sex

For CNS neoplasms (Fig. 4), the risk for South Asians was 25% lower than that of Whites. There was strong evidence of heterogeneity within the group with Pakistanis at lower risk than Indians (0.68 and 0.95 respectively; *p* = 0.005).

**Fig. 5:**
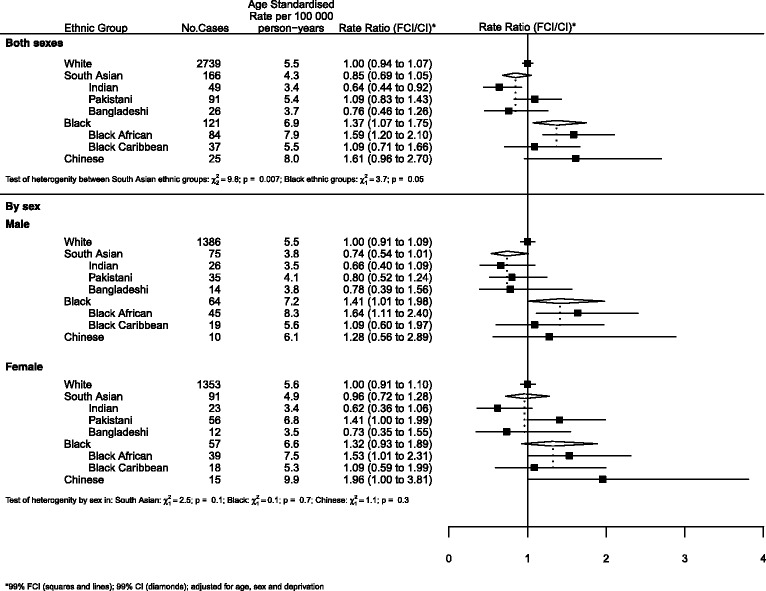
Other solid tumours by ethnicity and sex

For other cancers (Fig. 5), while the risk for South Asians was similar to Whites, there was evidence of heterogeneity within this group. Indians and Bangladeshis were at lower risk than Pakistanis (RRs = 0.64, 0.76 and 1.09 respectively; *p* = 0.007). The risk for Blacks was approximately 40% higher than Whites. There was some evidence of heterogeneity within this group with Black Africans at higher risk than Black Caribbeans (1.59 and 1.09 respectively; *p* = 0.05).

### Missing data and sensitivity analysis

For childhood cancers registered in the period 2001–2007, ethnicity from HES was 88% complete and from Cancer Registries it was 53% complete. The percentage of missing ethnicity data from HES that was supplemented by Cancer Registry data was 3%. Our missing ethnicity data as a whole ranged (for each cancer) from 7.0% – 11.8% (Table 2).

The incidence rate ratios for each (and all) cancer (Additional file 1: Figure S1) were very similar after sensitivity analyses (using multiple imputations of the missing ethnicity values based on age, sex, income and site of cancer).

## Discussion

Analysing nationwide data for childhood cancer, we have presented results in this paper that suggest an overall increased risk of childhood cancers in Pakistani and Black African children relative to White children. We were also able to further assess the major childhood cancers and their incidence within self-reported ethnic groups. Here, we confirmed the well described [24–28] increased risk of leukaemia and lymphoma in South Asian children, but for leukaemia also show this being due to the greater risk in Pakistani children in particular. In contrast to our findings in Indian children in Leicester, South Asian children appeared to have a lesser risk of CNS cancers. This has also been found in previous studies [24, 27, 29, 30], but these studies were underpowered and did not reach statistical significance, nor provide evidence for the lower risk of CNS cancers in Pakistani children compared to Indian children. Finally, an increased risk of ‘other solid tumours’ was observed in Black African children, likely driven by the previously described excess of renal tumours in this ethnic group [31, 32], though in this study we did not have sufficient numbers to estimate the relative risk.

We have previously discussed [12] how ethnicity is likely a proxy for genetic and/or environmental factors that might modify cancer risk, and how varying rates of cancers between ethnic groups may therefore be explicable through exploring the (differing) prevalence of putative risk/protective factors between ethnic groups. Where data for different ethnic groups could be found, some such factors are presented in Table 3, and discussed further below.

**Table 3:** Prevalence of some risk factors associated with childhood cancers, by ethnic group in the general population (most data sources: 2001–2010)

	Cancer Associated with Risk Factor^a^	British White	British Indian	British Pakistani	British Bangladeshi	British Black African	British Black Caribbean	British Chinese
Parental risk factors:								
Maternal age at pregnancy >35 years (%) [34, 56, 57]	Leukaemia; Lymphoma; CNS; Bone; Wilm’s	20	12	10	7	20	26	---
Maternal alcohol intake in pregnancy (%) [35, 56, 58]	Acute Myeloid Leukaemia	37	12	0	0	20	20	---
Maternal smoking in pregnancy (%) [36, 37, 56, 58]	Acute Myeloid Leukaemia; CNS	37	6	4	4	22	22	---
Epstein-Barr virus prevalence in pregnancy (%)^b^ [59]	Hodgkin’s Disease	94	*94*	*94*	*94*	---	---	---
Breastfed for at least four months (%) [58, 60, 61]	↓Acute Lymphoblastic Leukaemia	27	41	26	26	50	50	---
Paternal smoking (male prevalence 2003/4) [38, 39, 56, 62]	Acute Lymphoblastic Leukaemia	27	23	18	35	26	22	19
General Risk Factors:								
Household size (mean) [63, 64]	Leukaemia	2.3	3.4	4.3	4.3	2.6	2.2	---
High birth weight > 4000 g (%) [33, 56, 57]	All childhood cancers	13	3	5	3	9	6	---
HIV (%)^b c^ [43, 65]	Non-Hodgkin’s lymphoma	53	*1*	*1*	*1*	35	3	---
Epstein-Barr virus prevalence (%)^b d^ [66]	Hodgkin’s disease	44	*95*	*95*	*95*	---	---	---
Asthma prevalence (%) [52, 53]	↓CNS	32	24	29	26	23	33	---

^a^Direction of effect increased unless indicated with ↓

^b^Combined estimate (italicised) for some ethnic minority groups

^c^Proportional breakdown across ethnicities of diagnosed HIV infected adults seen for care in England, Wales and NI (2003)

^d^within a study of children with Hodgkin’s disease, diagnosed 1981–1999

--- Data not available

High birthweight has been associated with an increased risk of leukaemia (and possibly non-leukaemia cancers in older - ≥3 years old – children) [33]. Similarly, advancing maternal age has also been associated with a small increased risk (<10%) [34] in all groups of childhood cancer – leukaemia, lymphoma, CNS - analysed in this study. The above and other factors, such as maternal alcohol consumption in pregnancy [35], and maternal [36, 37] and paternal smoking [38, 39], all of which been shown to be associated with an increased childhood cancer risk (albeit inconsistently and to varying degrees for different cancers and subtypes) are all generally of greater prevalence in British Whites. Yet, our main findings are those of an increased cancer risk overall, and in leukaemias and lymphomas in particular, in some South Asian and the Black African ethnic minority groups.

As seen in Table 1, whilst a greater proportion of these groups in whom we observed a higher risk of leukaemias (Pakistanis) and lymphomas (South Asians and Black Africans) are from a lower income domain (and this study has adjusted for deprivation), recent large representative population based studies have not observed an association of deprivation with leukaemia or lymphoma subtypes [40, 41].

The relatively greater prevalence of HIV in Black Africans (in whom HIV exposure is mainly through sex between men and women [42]) is likely driving the increased risk of childhood lymphoma observed here and in other studies [43–45].

The one group of cancers in which a reduced risk relative to British Whites was observed (RR = 0.71) was in CNS cancers in South Asian children (apparently driven by a 32% lesser risk in Pakistani children). This finding is in keeping with previous UK studies (referenced above), many of which were in communities wherein there are large Pakistani populations, and showed a similar (though non-significant) reduced risk in South Asians relative to non-South Asians.

There are few well established risk factors for childhood CNS cancers [46–48]. Of these are a number of hereditary syndromes, which given the higher rate of consanguineous marriage in Pakistani families [49], one might expect a similarly increased risk of CNS cancers. However, such syndromes are thought likely to contribute to relatively few cases [50]. Asthma, or atopy more generally, is a more prevalent, proposed protective factor in CNS cancers [51, 52]. However, its prevalence does not appear to be markedly different across different ethnic groups [53] (Table 3).

This study has many of the strengths of our previous studies, namely the use of self-assigned ethnicity as a more accurate measure of ethnicity, as well as the same method being used for both numerator (Cancer Registry and HES) and denominator (Census), and the ability to separate large heterogeneous ethnic groupings (e.g. South Asian, Black) into more ethnically similar subgroups.

A further additional strength of this study relative to our analysis of childhood cancer in Indian and White children in Leicester was the use of national data, with this much greater sample size and number of outcomes allowing for greater power and precision in our estimates. We were able to adjust for age, sex and deprivation (all potential confounders in studying the association between ethnicity and cancers), and indeed also present results by sex. Using national data, where the method of ascertainment of cases is similar across the country also allows for a more accurate comparison between ethnic groups relative to those studies which compare rates of disease in groups in different countries. This is, to our knowledge, the first national study of childhood cancer incidence rate ratios between ethnic groups using self-assigned ethnicity. Additionally, there was little missing ethnicity data (Table 2) in these cancers, markedly lower than in our previous studies and other studies which have used HES data in combination with other methods [30].

Limitations remain however, in this being a population level study without information on individual exposures. Further, despite our presenting results by smaller, more homogenous ethnic subgroups, there remain within these groups a degree of heterogeneity, e.g. with Black Africans having a number of countries of origin, and similarly with Indians and Pakistanis originating from a number of provinces and states, with the cultural and genetic diversity that results in different ethnic groups.

As we have previously noted [6], we considered the ‘White’ classification to be ‘British White’ - though there would have been ‘Irish White’ and ‘Other White’ present in the ‘White’ classification (until 2003). However, these would have been very few (4% in the 2001 census), and unlikely therefore to have affected the results for British Whites. Finally, despite our use of self-assigned ethnicity as the current best measure of ethnicity, there remains a discordance – more so in ethnic minorities - between HES ethnicity recording and self-assigned ethnicity and there is an ongoing need to improve the accuracy of this data [54].

## Conclusions

Improvements in data collection and linkage of databases in recent years have permitted a more detailed and accurate study of ethnicity as a possible risk or protective factor in a number of different diseases. Initial descriptive studies such as this highlight associations between ethnicity as an exposure and outcomes such as childhood cancers; whilst it is not yet of course possible to draw conclusions regarding correlation, the awareness of these differences between ethnic groups based on high quality data allows for better public health planning and targeted initiatives, and the development of further research to aim to understand why these differences might exist, potentially giving rise to individual level, translational research [55].

## Additional file

Additional file 1: Figure S1. Sensitivity Analysis - Each cancer and all cancers by ethnicity, using imputed data. Sensitivity analyses for each cancer, and all cancers, by ethnicity (using multiple imputations of the missing ethnicity values based on age, sex, income and site of cancer). (PDF 3 kb)

## Abbreviations

(F)CI: (floating) confidence intervals
(I)RR: (incidence) rate ratios
ASR: Age-standardised rates
CNS: Central nervous system
CRUK: Cancer Research UK
HES: Hospital episodes statistics
HIV: Human immunodeficiency virus
ICCC-3: International classification of childhood cancer, 3rd edition
ICD-10: International classification of diseases, 10th revision
ICD-O-2/3: International classification of diseases of oncology, 2nd/3rd revisions
IMD 2007: Index of multiple deprivation 2007
NCIN: National cancer intelligence network
ONS: Office of National Statistics
