# Supplementary figures and images for: Childhood cancer incidence by ethnic group in England, 2001–2007: a descriptive epidemiological study
Source: BMC Cancer. 2017 Aug 25;17:570. doi: 10.1186/s12885-017-3551-7 (PMC5574126; doi:10.1186/s12885-017-3551-7)

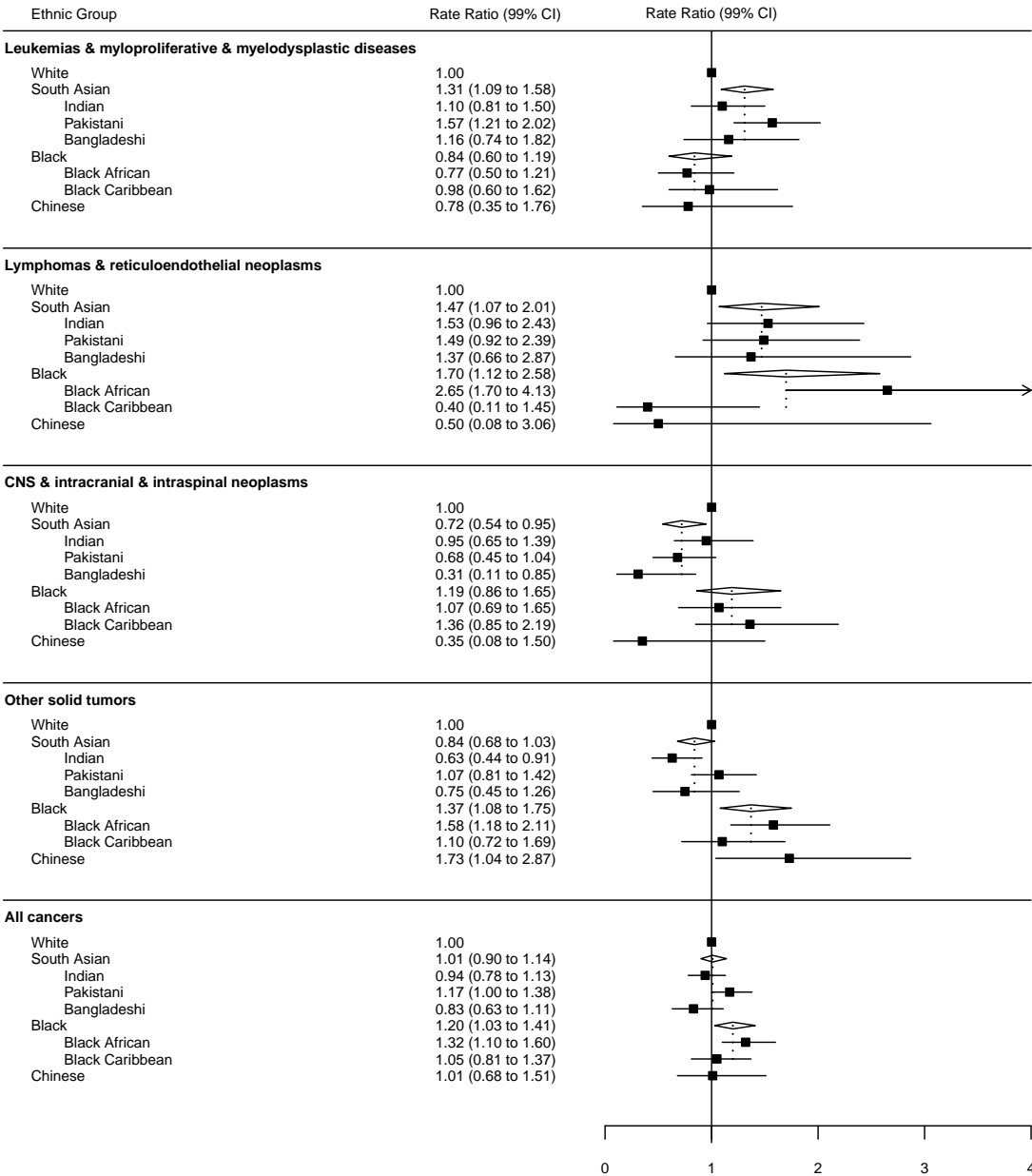

\*99% FCI (squares and lines); 99% CI (diamonds); adjusted for age, sex and deprivation

Supplement: Additional file 1: Figure S1. — Sensitivity Analysis - Each cancer and all cancers by ethnicity, using imputed data. Sensitivity analyses for each cancer, and all cancers, by ethnicity (using multiple imputations of the missing ethnicity values based on age, sex, income and site of cancer). (PDF 3 kb) [file 12885_2017_3551_MOESM1_ESM.pdf]
